# Intracellular Mycobacterium leprae Utilizes Host Glucose as a Carbon Source in Schwann Cells

**DOI:** 10.1128/mBio.02351-19

**Published:** 2019-12-17

**Authors:** Khushboo Borah, Karina do Carmo de Vasconcelos Girardi, Tom A. Mendum, Leticia Miranda Santos Lery, Dany J. V. Beste, Flavio Alves Lara, Maria Cristina Vidal Pessolani, Johnjoe McFadden

**Affiliations:** a Faculty of Health and Medical Sciences, University of Surrey, Guildford, United Kingdom; b Laboratório de Microbiologia Celular, Instituto Oswaldo Cruz, Rio de Janeiro, Brazil; University of Pittsburgh

**Keywords:** *Mycobacterium leprae*, Schwann cells, glucose, *Mycobacterium tuberculosis*, macrophage, phosphoenolpyruvate carboxylase, carbon metabolism

## Abstract

New approaches are needed to control leprosy, but understanding of the biology of the causative agent Mycobacterium leprae remains rudimentary, principally because the pathogen cannot be grown in axenic culture. Here, we applied ^13^C isotopomer analysis to measure carbon metabolism of M. leprae in its primary host cell, the Schwann cell. We compared the results of this analysis with those of a related pathogen, Mycobacterium tuberculosis, growing in its primary host cell, the macrophage. Using ^13^C isotopomer analysis with glucose as the tracer, we show that whereas M. tuberculosis imports most of its amino acids directly from the host macrophage, M. leprae utilizes host glucose pools as the carbon source to biosynthesize the majority of its amino acids. Our analysis highlights the anaplerotic enzyme phosphoenolpyruvate carboxylase required for this intracellular diet of M. leprae, identifying this enzyme as a potential antileprosy drug target.

## Abstract (precis)

Leprosy remains a major problem in the world today, particularly affecting the poorest and most disadvantaged sections of society in the least developed countries of the world. The long-term aim of research is to develop new treatments and vaccines, and these aims are currently hampered by our inability to grow the pathogen in axenic culture. In this study, we probed the metabolism of M. leprae while it is surviving and replicating inside its primary host cell, the Schwann cell, and compared it to a related pathogen, M. tuberculosis, replicating in macrophages. Our analysis revealed that unlike M. tuberculosis, M. leprae utilized host glucose as a carbon source and that it biosynthesized its own amino acids, rather than importing them from its host cell. We demonstrated that the enzyme phosphoenolpyruvate carboxylase plays a crucial role in glucose catabolism in M. leprae. Our findings provide the first metabolic signature of M. leprae in the host Schwann cell and identify novel avenues for the development of antileprosy drugs.

## OBSERVATION

Leprosy is an ancient infectious disease that remains a major cause of chronic morbidity in vulnerable populations in developing countries, despite the availability of effective, though very lengthy, treatment (1, 2). Victims continue to be stigmatized such that the disease has recently been described as “the world’s oldest human-rights issue” (3). New approaches are needed to control the disease, such as effective vaccines and shorter treatment regimens (2, 4, 5). Research into leprosy, however, is severely hampered by our inability to grow the causative agent, Mycobacterium leprae, in axenic culture (6). Genome sequencing revealed that the M. leprae genome is drastically reduced compared to the Mycobacterium tuberculosis genome (7), suggesting that the organism’s obligate intracellular lifestyle has driven the loss of genes that are dispensable in its human host, and potentially rendering its replication dependent on nutrients available only *in vivo*. However, despite this genomic downsizing, its essential central metabolic pathways appear to remain intact and as competent as those of M. tuberculosis, so why M. leprae fails to grow *in vitro* remains a puzzle. Also, there remains a major question about the intracellular nutrient sources utilized by M. leprae growing inside its human host cell. It is increasingly recognized that metabolism plays a key role in the survival and virulence of intracellular pathogens (8–11). Carbon metabolism has been extensively investigated in M. tuberculosis both *in vitro* (9, 10) and *ex vivo* (8, 11) as a route toward identification of novel drug targets. It has been shown that M. tuberculosis accesses multiple carbon sources, including lipids and cholesterol, when replicating inside its host macrophage cell (8, 10, 11). For intracellular M. leprae, impairment of host cell cholesterol metabolism decreases its survival, but cholesterol was not utilized as either an energy or carbon source by the bacilli (12–17). This is consistent with *in silico* studies that demonstrated that M. leprae has lost many genes for cholesterol catabolism, including the Mce4 operon, which codes for a sterol lipid transport system found in other mycobacteria, including M. tuberculosis (12). Fatty acids have also been suggested as potential carbon sources, as palmitic acid is oxidized by M. leprae (18, 19). Infection by M. leprae increases glucose uptake in Schwann cells (20), yet whether this relates to the uptake of glucose as a carbon source by M. leprae is unknown.

Here, ^13^C isotopomer analysis was used to study the metabolic interactions between M. leprae and Schwann cells, and these results were compared to the profile of M. tuberculosis replicating within human macrophages. Our analysis shows major differences in the metabolic adaptations of these two pathogens in their respective intracellular environment.

## 

### Assimilation of [^13^C_6_]glucose by host and pathogen.

**FIG 1:**
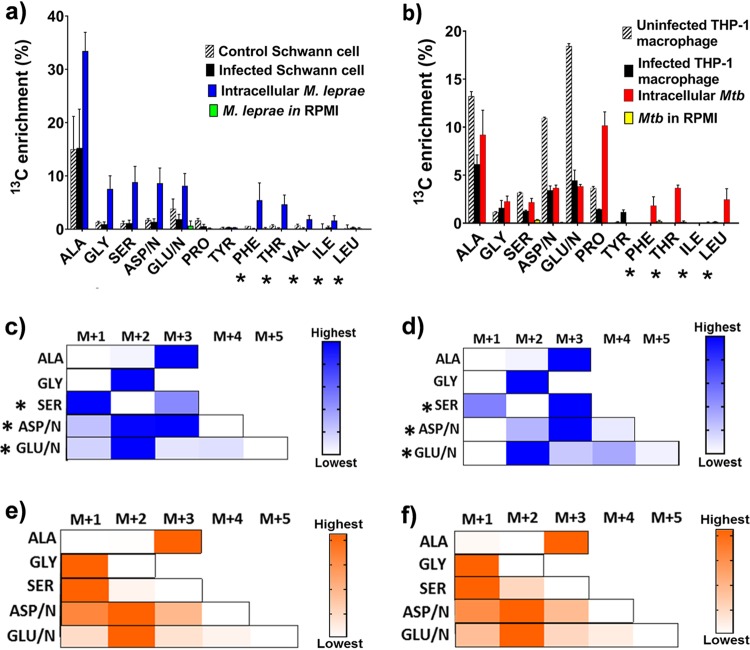
Assimilation of [^13^C_6_]glucose by pathogens and host cells. (a and b) Average ^13^C enrichments are compared between uninfected Schwann cells, M. leprae-infected Schwann cells, intracellular M. leprae, and M. leprae in RPMI 1640 medium (control) (a) and uninfected THP-1 macrophage, M. tuberculosis-infected THP-1 macrophages, intracellular M. tuberculosis (*Mtb*), and *Mtb* in RPMI 1640 medium (control) (b). (c to f) ^13^C isotopomer profiles are shown in M. leprae-infected Schwann cells (c), intracellular M. leprae (d), M. tuberculosis-infected THP-1 macrophages (e), and intracellular M. tuberculosis (f). Measurements are shown as heat maps with a single gradient to highlight the proportional abundances of isotopomers for an amino acid. M+1, M+2, M+3, M+4, and M+5 are the isotopomer families with different numbers of labeled carbons. Significant differences in the profiles between M. leprae and infected Schwann cells are indicated by an asterisk. The relative abundances of each isotopomer (see Data Sets S2 and S3 in the supplemental material) are indicated by a color gradient. Amino acids and *m*/*z* values are as follows: alanine (ALA), *m/z* 260; glycine (GLY), *m/z* 246; serine (SER), *m/z* 390; aspartate/asparagine (ASP/ASN [ASP/N]), *m/z* 418; glutamate/glutamine (GLU/GLN [GLU/N]), *m/z* 432; phenylalanine (PHE), *m/z* 336; threonine (THR), *m/z* 404; valine (VAL), *m/z* 288; proline (PRO), *m/z* 258; isoleucine (ILE), *m/z* 274; and tyrosine (TYR), *m/z* 466. Amino acids not shown in the panels had no detectable ^13^C. Essential amino acids in Schwann cells (a) and THP-1 macrophages (b) are marked with an asterisk. Measurements are averages plus standard deviations (SD) (error bars) from three independent infection experiments and are included in Data Sets S2 and S3.

Previous studies showed that infection with M. leprae boosted the glucose uptake rate of Schwann cells, suggesting that this sugar is a potential carbon source for the pathogen (20). To test this hypothesis, Schwann cells were infected with M. leprae in the presence of [^13^C_6_]glucose. We used a multiplicity of infection (MOI) of 100:1 in order to obtain approximately 83% of Schwann cells infected with M. leprae (14). Infected cells were then incubated in [^13^C_6_]glucose-containing tissue culture media, before they were harvested, lysed, and separated into eukaryotic and bacterial fractions using our previously established methods (8). To directly compare results to the intracellular metabolism of M. tuberculosis, we performed the same postinfection [^13^C_6_]glucose labeling experiments with the THP-1 macrophage-M. tuberculosis model, which served as a control for our M. leprae-Schwann cell model (8). Control experiments were performed using uninfected mammalian cells (Schwann or macrophages) and bacteria (M. tuberculosis or M. leprae) incubated in RPMI 1640 medium containing [^13^C_6_]glucose. Cells were lysed, and ^13^C enrichment (^13^C incorporated from the tracer) and isotopomer distribution of amino acids were measured in eukaryotic and bacterial fractions by gas chromatography-mass spectrometry (GC-MS) (21, 22) (Fig. 1; see also Data Set S1 in the supplemental material). As an additional check, we also performed a prelabeling experiment, in which Schwann cells were passaged three times in [^13^C_6_]glucose-containing medium prior to infection with M. leprae, followed by recovery and ^13^C analysis of host and bacterial amino acids. However, by using this experimental approach, we found significantly lower levels of ^13^C incorporation with only a few amino acids labeled in M. leprae. This was probably because of dilution of label in prelabeled Schwann cells (see Fig. S1, panel i.a in the supplemental material). The comparison of ^13^C profiles in the detected amino acids of M. leprae from the prelabeling experiment were identical to those derived from postinfection pulsed Schwann cells (Fig. S1, panel ii). This confirms that the labeling profiles from pulsed experiments were not biased by the experimental approach used in this study.

10.1128/mBio.02351-19.1 DATA SET S1 ^3^C enrichments measured in infected and uninfected Schwann cells, intracellular M. leprae and M. leprae in RPMI 1640 medium, infected and uninfected THP-1 macrophages, intracellular M. tuberculosis (*Mtb*) and *Mtb* in RPMI 1640 medium. Download Data Set S1, XLSX file, 0.01 MB. Copyright © 2019 Borah et al. 2019 Borah et al. This content is distributed under the terms of the Creative Commons Attribution 4.0 International license.

10.1128/mBio.02351-19.4 FIG S1 (i) ^13^C incorporation in Schwann cells (infected and uninfected) and intracellular M. leprae. Measurements are shown for host and bacteria from prelabeled setup (a) and pulse experimental setup (b). In the prelabeled setup, Schwann cells were grown in ^13^C-labeled RPMI medium before infection with M. leprae. For pulse experiments, Schwann cells were infected with M. leprae, followed by incubation of infected cells in ^13^C-labeled medium. Data for prelabeled Schwann cell experiment are shown for a single infection and are the average ± standard deviations (SD) (error bars) from three independent experiments. (ii) Mass isotopomer distribution of amino acids in intracellular M. leprae isolated from prelabeled and pulse-labeled Schwann cells. Amino acid profiles are plotted on a metabolic map showing the reactions for the TCA cycle, glycolysis, PPP, and anaplerotic pathway (ANA). M+1, M+2, M+3, M+4, M+5, M+6, M+7, M+8, and M+9 are the mass isotopomer families. Proportional increases or decreases in the ^13^C abundance of mass isotopomers of a metabolite are indicated as heat maps with a single gradient. The isotopomer family occupying the highest proportion of ^13^C is shown with the highest color intensity. Download FIG S1, TIF file, 0.2 MB. Copyright © 2019 Borah et al. 2019 Borah et al. This content is distributed under the terms of the Creative Commons Attribution 4.0 International license.

To validate our results, it was first necessary to demonstrate that the fractionation protocol successfully separated host and bacterial cells. This was confirmed by the demonstration that intracellular M. leprae and infected Schwann cell fractions had different ^13^C enrichment of amino acids (Fig. 1a). Significantly, there was no ^13^C label incorporated the essential amino acids of Schwann cells, such as phenylalanine (Phe), threonine (Thr), valine (Val), and isoleucine (Ile), but label was incorporated into these amino acids derived from the bacterial fraction, confirming the separation of Schwann cell and M. leprae compartments. This finding also demonstrated that intracellular M. leprae bacilli are metabolically active and synthesizing new protein (Fig. 1a and Data Set S1). In contrast, there was very little ^13^C incorporation when M. leprae was incubated in RPMI 1640 medium, consistent with the lack of axenic growth (Fig. 1a). These control experiments also confirmed that the incorporation of label into M. leprae is taking place within its host cell, rather than in the RPMI 1640 tissue culture medium, confirming that the intracellular results represented the metabolism of M. leprae within its host cell, rather than being an artifact of being in a pool of RPMI 1640 medium. For M. tuberculosis-infected THP-1 macrophages, we observed a similar pattern of segregation of ^13^C assimilation as previously described (8) with no enrichment in the essential amino acids of the host cell fractions, but the same amino acids were labeled in the M. tuberculosis’s fraction (Fig. 1b).

The incorporation of ^13^C from [^13^C_6_]glucose into M. leprae amino acids demonstrates that the leprosy bacillus has access to glucose-derived carbon from the host cell. The majority of the amino acids showed <10% ^13^C enrichment. This is likely to be a reflection of the slow dynamics of replacement of unlabeled amino acid pools synthesized before incubation with labeled glucose. It may also reflect utilization of additional unlabeled carbon sources. However, considering only the ^13^C enrichment data, it is not possible to distinguish whether glucose or some product of glucose catabolism (such as pyruvate) is imported from the host cell. To gain further insights into the precise nature of the source of carbon, we first needed to ascertain which amino acids were imported directly from the host cell and which were biosynthesized by M. leprae and thereby function as reporters of M. leprae metabolism. We compared the ^13^C mass isotopomer distribution (the pattern and proportion of ^12^C/^13^C in the carbon backbone) of five amino acids that had significant (>1%) ^13^C enrichment in both hosts and the pathogens. The profiles of three M. leprae amino acids, serine (Ser), aspartate/asparagine (Asp/Asn), and glutamate/glutamine (Glu/Gln), were significantly different from the same amino acids from the infected Schwann cell (Fig. 1c and d), indicating that these amino acids are biosynthesized by the pathogen rather than imported from the host.

Of the labeled M. leprae amino acids, only alanine (Ala) and glycine (Gly) had similar isotopomer profiles in M. leprae and in Schwann cells, suggesting that these amino acids could be acquired by M. leprae directly from the host cell (Fig. 1c and d). In contrast, the profiles of all five of these amino acids from intracellular M. tuberculosis were indistinguishable from the same amino acids in its host THP-1 macrophage (Fig. 1e and f), indicating that, as previously described (8), M. tuberculosis imports these amino acids from its host cell and directly incorporates them into biomass. Our finding that M. leprae amino acids are biosynthesized rather than imported is consistent with previous proteomic analyses, which showed that enzymes required for biosynthesis of several amino acids such as Asp/Asn, Ala, Thr, cysteine, proline (Pro), lysine, histidine, leucine, Ile, and Val are synthesized by intracellular M. leprae (23).

### M. leprae utilized host glucose pools to biosynthesize amino acids.

**FIG 2:**
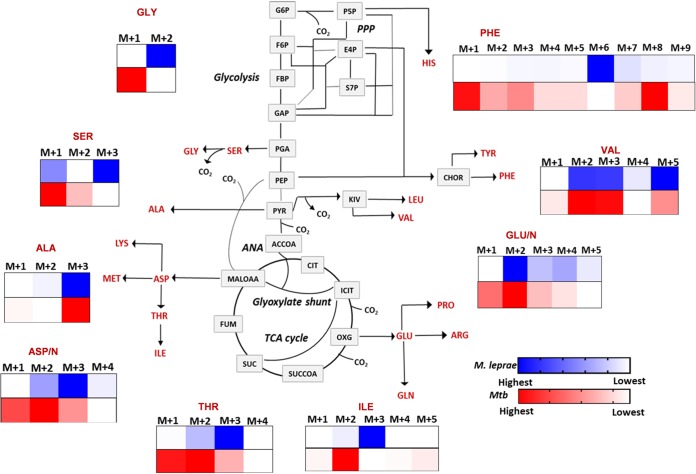
^13^C isotopomer profiles of amino acids in intracellular M. leprae versus M. tuberculosis (*Mtb*). (A) Amino acid profiles are plotted on a metabolic map showing the reactions for the TCA cycle, glycolysis, PPP, and anaplerotic pathway (ANA). M+1, M+2, M+3, M+4, M+5, M+6, M+7, M+8, and M+9 are the mass isotopomer families. Proportional increases or decreases in the ^13^C abundance of mass isotopomers of a metabolite are indicated by a single gradient. The isotopomer family occupying the highest proportion of ^13^C is shown with the highest color intensity. The metabolites are malate oxaloacetate (MALOAA), fumarate (FUM), succinate (SUC), succinyl coenzyme A (SUCCOA), 2-oxoglutarate (OXG), isocitrate (ICIT), citrate (CIT), acetyl coenzyme A (ACCOA), pyruvate (PYR), phosphoglyceric acid (PGA), glyceraldehyde-3-phosphate (GAP), fructose-6-bisphosphate (FBP), fructose-6-phosphate (F6P), glucose-6-phosphate (G6P), pentose-5-phosphate (P5P), erythrose-4-phsophate (E4P), sedoheptulose-7-phosphate (S7P), ketoisovalerate (KIV), chorismate (CHOR), histidine (HIS), glutamine (GLN), glutamic acid (GLU), lysine (LYS), methionine (MET), and arginine (ARG). Measurements for M. leprae and M. tuberculosis are averages ± SD from three independent infection experiments (Data Sets S1 and S2). Statistically significant changes (*P* < 0.05) between the quantitative proportions of M. leprae and M. tuberculosis obtained by Student’s *t* test are indicated by an asterisk.

Since amino acids are synthesized from precursors in central metabolism, the isotopomer profile of biosynthesized (rather than imported) amino acids is a reporter of M. leprae’s central carbon metabolism. For amino acids such as Val, Gly, and Ser that are synthesized from precursors in the glycolytic arm of metabolism, all carbon atoms were ^13^C labeled in intracellular M. leprae, indicating that the entire ^13^C_6_ backbone of the labeled glucose was delivered intact to their precursor (Fig. 2 and Fig. S2). This was in contrast to the pattern for M. tuberculosis, in which only Ala was fully labeled, whereas Val, Ser, and Gly had fewer labeled carbon atoms, indicating considerable carbon shuffling between the carbon backbone of labeled glucose and their precursor. The labeling pattern for Phe, which is synthesized from both the glycolytic and pentose phosphate pathway (PPP) arms of the metabolic network, was also very different between M. leprae and M. tuberculosis. In particular, the highest ^13^C isotopomer for Phe in M. leprae was M+6 (six carbon atoms are ^13^C labeled), indicating that the pathogen incorporates the entire carbon backbone of glucose into its biosynthesis (Fig. 2 and Fig. S2). In contrast, Phe in M. tuberculosis was characterized by a range of isotopomers with a notable absence of M+6, consistent with considerable carbon shuffling between labeled glucose and the amino acid’s precursors and consistent with previous studies that indicate that pathogen imports carbon substrates, other than glucose, such as lipids, from its host cell (8, 10, 11, 24).

10.1128/mBio.02351-19.5 FIG S2 Comparison of mass isotopomer distributions in intracellular M. leprae versus intracellular M. tuberculosis (*Mtb)*. M+1, M+2, M+3, M+4, M+5, M+6, M+7, M+8, and M+9 are the mass isotopomer families. Statistically significant changes (*P* < 0.05) between the quantitative proportions of M. leprae and *Mtb* obtained by Student’s *t* test are indicated by an asterisk. Download FIG S2, TIF file, 0.2 MB. Copyright © 2019 Borah et al. 2019 Borah et al. This content is distributed under the terms of the Creative Commons Attribution 4.0 International license.

The amino acids derived from the tricarboxylic acid cycle (TCA) cycle, including Asp/Asn, Thr, and Ile, also showed very different profiles between M. leprae and M. tuberculosis (Fig. 2 and Fig. S2). For M. tuberculosis, the predominant isotopomers were M+1 and M+2, consistent with our previous studies indicating that the intracellular pathogen utilizes carbon sources, such as host-derived lipids (rather than glucose), which are catabolized through the oxidative TCA cycle (8, 9). A recent work by Serafini et al. (25) also demonstrated that M. tuberculosis utilized both lactate and pyruvate as carbon sources *in vitro*, suggesting that these terminal glycolytic intermediates could be carbon sources for M. tuberculosis during infection. In contrast, the predominant isotopomer for these amino acids in M. leprae was M+3, suggesting that the carbon backbone of their precursor, oxaloacetate, is derived from glucose via anaplerotic carboxylation of phosphoenolpyruvate (PEP) by phosphoenolpyruvate carboxylase (7). The ^13^C isotopomer profile for Asp/Asn from prelabeling experiments was also similar, demonstrating identical pattern of glucose utilization via phosphoenolpyruvate carboxylase in both experiments (Fig. S1, panel ii).

### Infection induces metabolic perturbations in the host cell.

We compared the ^13^C isotopomer profiles of amino acids in uninfected (control) and infected Schwann cells and THP-1 macrophages (Fig. S3a to g). There were no significant alterations in the profiles of Ala, Gly, Ser, Asp/Asn, Glu/Gln, and Pro in infected Schwann cells, suggesting that there were no changes in host cell carbon flux through glycolysis and the TCA cycle (Fig. S3, panels a.i, b.i, c.i, d.i, e.i, and f.i) as a result of infection. This was in contrast to M. tuberculosis-infected THP-1 macrophages, which demonstrated differences in the metabolic profiles of Gly, Ser, Asp/Asn, Glu/Gln, and Pro amino acids that are derived from glycolysis and the TCA cycle (Fig. S3, panels b.ii, c.ii, d.ii, e.ii, and f.ii), suggesting major shifts in carbon flux through these host cell pathways upon M. tuberculosis infection. Host cell metabolism was not, however, entirely unperturbed by M. leprae infection. The isotopomer profile of tyrosine (Tyr) was different between uninfected and infected Schwann cells (Fig. S3, panel g.i). The carbon backbone of Tyr is derived from chorismate which is synthesized by the combination of carbons from erythrose-4-phosphate and two molecules of PEP that enter the PPP. The data suggest that M. leprae infection stimulates increased carbon flux into the PPP in Schwann cells. These findings are consistent with previous evidence of increased glucose-6-phosphate dehydrogenase activity, a key enzyme of the oxidative PPP, in infected Schwann cells (20). M. tuberculosis infection of macrophages also induced significant changes in the isotopomer profile of Tyr, indicating that infection with M. tuberculosis induced a similar increased routing of carbon flux through the PPP of host cells (Fig. S3, panel g.ii).

10.1128/mBio.02351-19.6 FIG S3 Changes in ^13^C isotopomer profiles of amino acids in Schwann cells and THP-1 macrophages upon infection. Data are shown for uninfected (control) versus infected Schwann cells in panels a.i to g.i and uninfected (control) versus infected THP-1 macrophages in panels a.ii to g.ii. Measurements for Schwann cells and THP-1 macrophages are average ± SD from three independent infection experiments. Download FIG S3, TIF file, 0.3 MB. Copyright © 2019 Borah et al. 2019 Borah et al. This content is distributed under the terms of the Creative Commons Attribution 4.0 International license.

**FIG 3:**
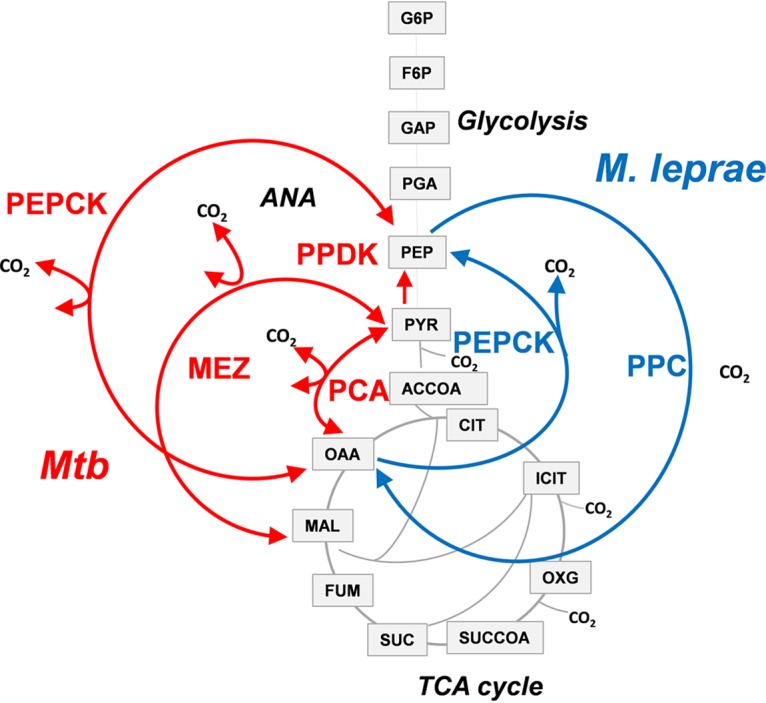
Anaplerotic nodes of M. leprae versus M. tuberculosis (*Mtb*). Enzymes of the node are PEP carboxylase (PPC), PEP carboxykinase (PEPCK), malic enzyme (MEZ), pyruvate phosphate dikinase (PPDK), and pyruvate carboxylase (PCA). Metabolites of glycolysis, TCA cycle, and ANA (anaplerotic node) are malate (MAL), oxaloacetate (OAA), fumarate (FUM), succinate (SUC), succinyl coenzyme A (SUCCOA), 2-oxoglutarate (OXG), isocitrate (ICIT), citrate (CIT), acetyl coenzyme A (ACCOA), pyruvate (PYR), phosphoglyceric acid (PGA), glyceraldehyde-3-phosphate (GAP), fructose-6-phosphate (F6P), and glucose-6-phosphate (G6P).

In summary, we demonstrated that the intracellular metabolism of M. leprae differs from that of M. tuberculosis. Unlike M. tuberculosis, M. leprae accesses host cell glucose pools as carbon sources and uses the anaplerotic pathway for the synthesis of amino acids derived from the TCA cycle. The primary M. leprae host cell, the Schwann cell, is the most important glial cell involved in metabolism and function of the nervous system, and Schwann cells have access to glucose as the primary energy source in the nervous system (26). Our data suggest that M. leprae accesses host glucose pools and possibly the structural analogs of glucose, such as fructose or galactose, or even a glucose (or isomer) polymer, such as glycogen, which is available in Schwann cells, potentially explaining the lack of glucose incorporation in RPMI 1640 medium (27). Note also that these differences in intracellular metabolism between M. leprae and M. tuberculosis are not predictable by considering the gene repertoire of these related pathogens, as despite the genetic downsizing of M. leprae, both pathogens encode all the genes necessary for the central metabolic pathways. However, in contrast to M. tuberculosis’s complex anaplerotic node which is composed of phosphoenolpyruvate carboxykinase (PEPCK), malic enzyme (MEZ), pyruvate carboxylase (PCA), and pyruvate phosphate dikinase (PPDK), the M. leprae genome encodes only two anaplerotic enzymes linking glycolysis and the TCA cycle: PEP carboxylase (PPC) and PEPCK (Fig. 3) (28, 29). In other bacterial species where both enzymes are present, such as Escherichia coli, Bacillus subtilis and Corynebacterium glutamicum, PEPCK is used primarily for gluconeogenesis, whereas PPC is employed for anaplerotic synthesis of oxaloacetate during glucose metabolism (30–32). Also, in E. coli, PEPCK failed to complement for the loss of PPC (30), suggesting that, in M. leprae, PPC plays an anaplerotic role. Our analysis thereby suggests that PPC is likely to be essential for the intracellular survival of M. leprae, and since it is absent in humans, it is a potential drug target for treatment of leprosy.

### Experimental procedures.

**(i) Bacterial strains and growth.** M. leprae Thai-53 strain of M. leprae was obtained from the Department of Biology at Lauro de Souza Lima Institute by Patricia Sammarco Rosa. Briefly, M. leprae was isolated from athymic *nu*/*nu* mouse footpads and then purified and counted at the Oswaldo Cruz Institute as described previously (33). For the experiments, M. leprae viability over 80% was measured by using a LIVE/DEAD bacterial viability kit (Life Technologies). M. tuberculosis H37Rv was cultivated on Middlebrook 7H11 agar and Middlebrook 7H9 broth supplemented with 5% (vol/vol) oleic acid-albumin-dextrose-catalase enrichment supplement (Becton Dickinson) and 0.5% (vol/vol) glycerol at 37°C with agitation (150 rpm).

**(ii) Schwann cell culture and M. leprae infection.** The human Schwann cell line ST88-14 was obtained from the American Type Culture Collection (ATCC) and the cell culture was maintained in RPMI 1640 medium supplemented with 10% fetal bovine serum (Cripion Biotechnology) in 5% CO_2_ atmosphere at 37°C. For infection, 3 × 10^6^ Schwann cells were incubated using a multiplicity of infection (MOI) of 100 bacteria per cell (100:1) for 48 h at 33°C, the ideal temperature of M. leprae maintenance. After 48 h of the infection, the medium was changed to 30 ml RPMI 1640 medium without glucose supplemented with 100% [U-^13^C_6_] labeled glucose (Cambridge Isotopes Laboratories) and 10% fetal bovine serum for 72 h.

**(iii) THP-1 cell culture and M. tuberculosis infection.** The THP-1 human monocytic cell line was obtained from ATCC TIB-202 and was cultured as previously described (8). Briefly, cells were grown in RPMI 1640 medium supplemented with 10% heat-inactivated fetal calf serum (Sigma). Macrophages were generated by differentiation of monocytes using 50 nM phorbol 12-myristate 13-acetate (PMA) (Sigma) for 72 h at 37°C, 5% CO_2_, and 95% humidity, and were used for infection assays. Bacterial infections were performed as previously described (8). Each flask was seeded with 1 × 10^6^ THP-1 cells, and differentiated cells were washed with phosphate-buffered saline (PBS) supplemented with 0.49 mM Mg^2+^ and 0.68 mM Ca^2+^ (PBS+ [PBS supplemented with CaCl_2_ and MgCl_2_]). M. tuberculosis cultures were grown exponentially in Middlebrook 7H9 liquid medium to an optical density of 1.0 (1 × 10^8^ CFU ml^−1^) for the infection and then washed in PBS and resuspended in RPMI 1640 medium. A total of 1 ml of bacterial suspension was added to each flask to achieve a MOI of 5 and incubated for 3 to 4 h. After incubation, the macrophages were washed three times with PBS+ (Sigma-Aldrich), and 30 ml of RPMI 1640 medium containing 100% [U-^13^C_6_] glucose (Cambridge Isotope Laboratories) was added to each flask and incubated for 48 h at 37°C and 5% CO_2_.

**(iv) [U-^13^C_6_]glucose labeling of bacterial cultures in RPMI 1640 medium.** M. leprae bacilli (3 × 10^8^) were maintained in RPMI 1640 medium containing 100% [U-^13^C_6_]glucose at 33°C for 48 h. M. tuberculosis bacilli (1 × 10^8^) were maintained in RPMI 1640 medium containing 100% [U-^13^C_6_]glucose at 37°C, 150 rpm for 48 h. After incubation, bacterial cultures were centrifuged at 11,000 × *g* for 10 min, and the amino acid extract was prepared as previously described (8), proceeding as summarized below.

**(v) Amino acid extraction.** Infected Schwann cells and macrophages were harvested by removing the culture medium, and the adhered cells were washed with 3 ml of ice-cold PBS and lysed with 0.1% Triton X-100 (8). The cellular and bacterial fractions for both infection models were harvested by differential centrifugation method (8). The bacterial pellet was washed twice with RIPA buffer (radioimmunoprecipitation assay buffer) (Sigma), and both bacterial and soluble amino acids from the cellular compartment were subjected to hydrolysis with 6 N hydrochloric acid (HCl) at 100°C overnight. After acid hydrolysis, samples were dried with nitrogen gas, followed by the addition of 1 ml of distilled water. Then, the samples were transferred to another tube and centrifuged at 11,000 × *g*. After centrifugation, samples were dried using nitrogen gas.

**(vi) ^13^C mass isotopomer analysis.** Amino acid hydrolysates were dried and derivatized using pyridine and *tert*-butyldimethyl silyl chloride (TBDMSCl) (Sigma) (21). Amino acids were analyzed using a VF-5ms inert 5% phenyl-methyl column (Agilent Technologies) on a gas chromatography-mass spectrometry (GC-MS) system. Due to hydrolysis, amino acid pairs aspartate/asparagine and glutamine/glutamate were detected together as a single pool in MS analysis. MS data were extracted using Chemstation GC-MS software (Agilent Technologies) and were baseline corrected using Metalign (22). Mass isotopomer data were corrected for natural isotope effects using MSCorr program (34). Average ^13^C in an amino acid was calculated from the fractional abundance of the ^13^C mass isotopomer in the entire fragment (21). Graphical representation and statistical analysis of the data were performed by using GraphPad Prism 8.0.

10.1128/mBio.02351-19.2 DATA SET S2 ^13^C mass isotopomer distribution of amino acids in uninfected Schwann cells, infected Schwann cells and intracellular M. leprae. Download Data Set S2, XLSX file, 0.01 MB. Copyright © 2019 Borah et al. 2019 Borah et al. This content is distributed under the terms of the Creative Commons Attribution 4.0 International license.

10.1128/mBio.02351-19.3 DATA SET S3 ^13^C mass isotopomer distribution of amino acids in uninfected THP-1 control macrophages, infected THP-1 macrophages and intracellular M. tuberculosis (*Mtb*). Download Data Set S3, XLSX file, 0.01 MB. Copyright © 2019 Borah et al. 2019 Borah et al. This content is distributed under the terms of the Creative Commons Attribution 4.0 International license.
