# Supplementary figures and images for: Intracellular Mycobacterium leprae Utilizes Host Glucose as a Carbon Source in Schwann Cells
Source: mBio. 2019 Dec 17;10(6):e02351-19. doi: 10.1128/mBio.02351-19 (PMC6918074; doi:10.1128/mBio.02351-19)

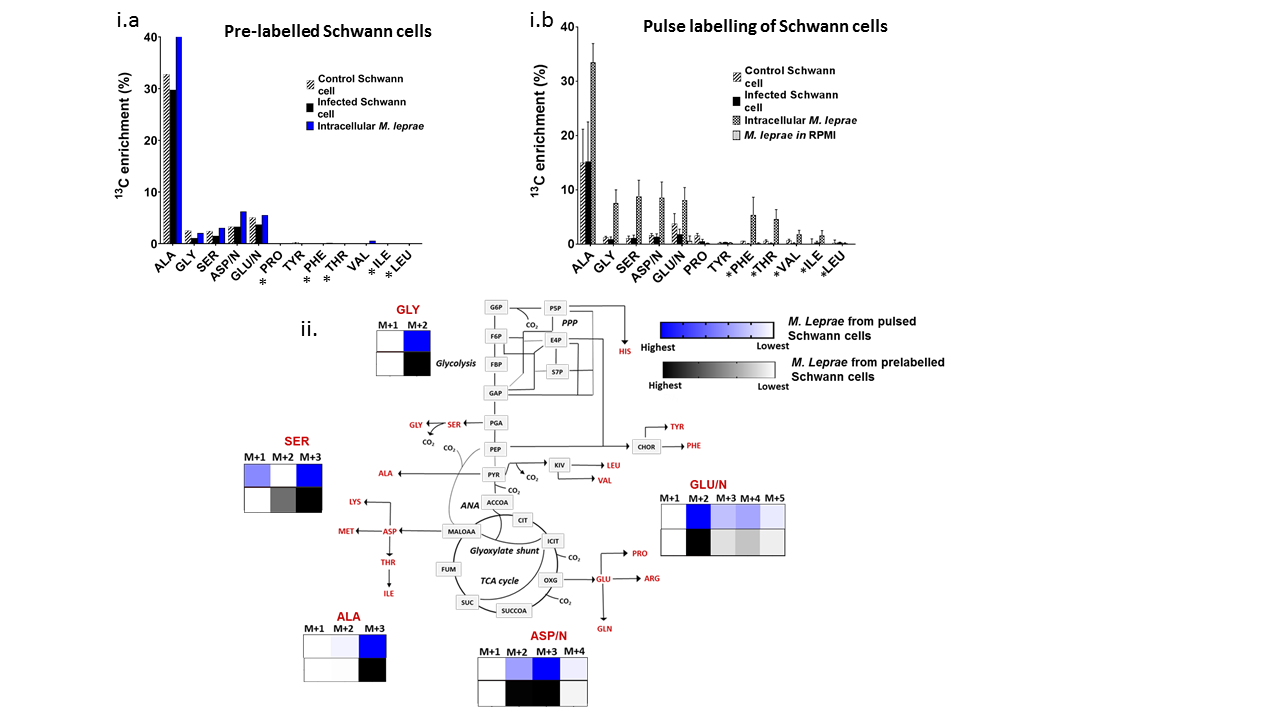

Supplement: FIG S1 [file mBio.02351-19-sf001.tif]

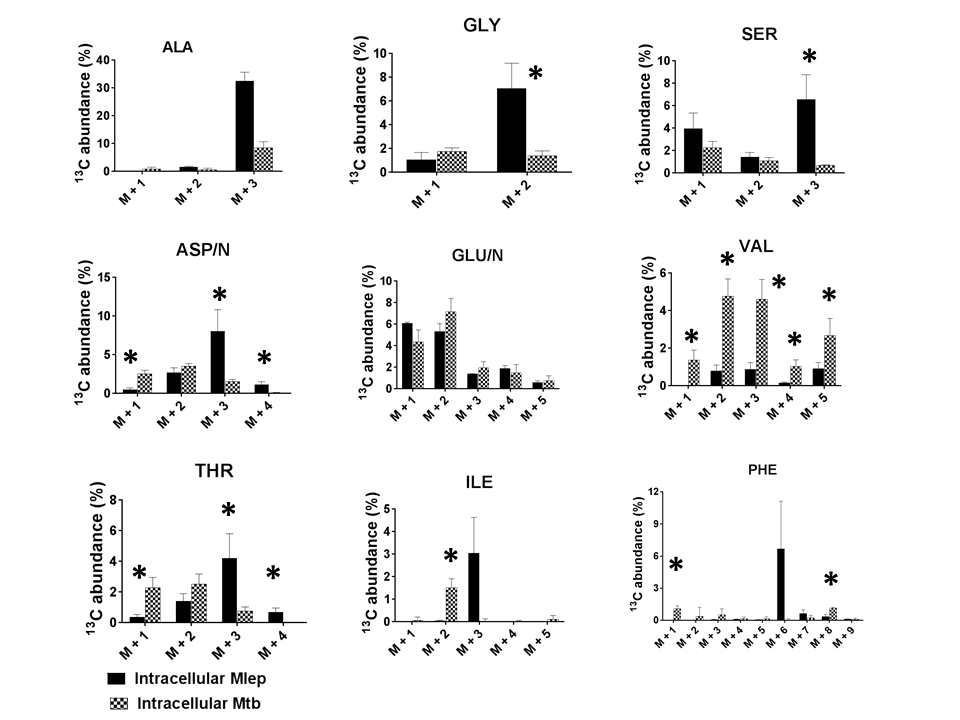

Supplement: FIG S2 [file mBio.02351-19-sf002.tif]

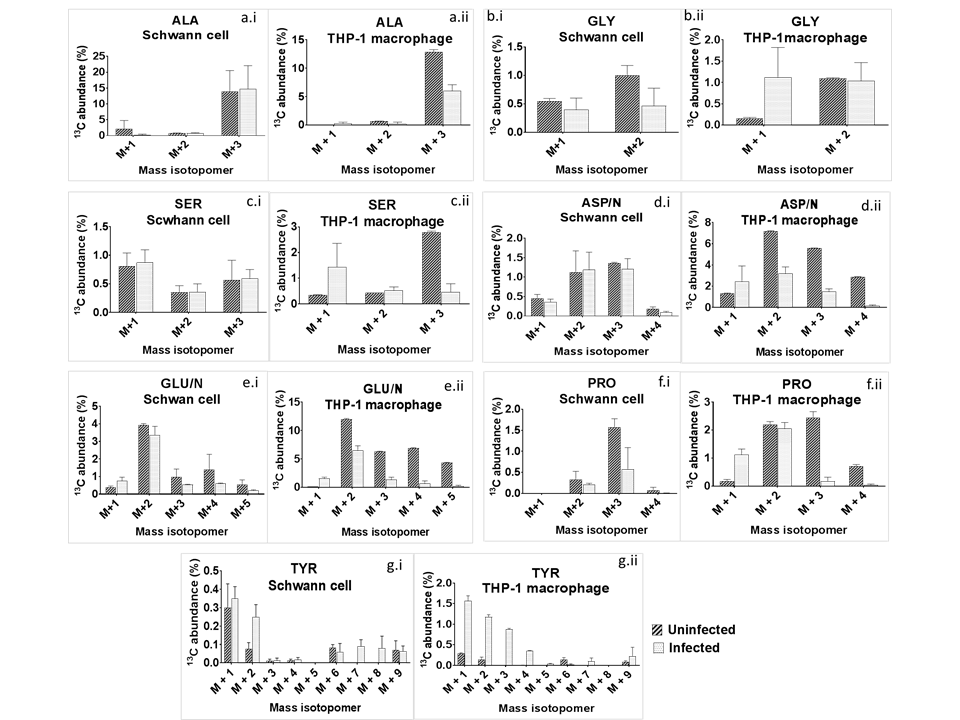

Supplement: FIG S3 [file mBio.02351-19-sf003.tif]
